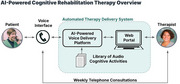# Improving Access to Dementia Care through AI‐Powered Cognitive Rehabilitation Therapy

**DOI:** 10.1002/alz70858_098721

**Published:** 2025-12-25

**Authors:** Jennifer A Flexman, Jessie Ng, Paul A Campbell, Rebecca Vaughan Darwish, Susan Kane

**Affiliations:** ^1^ Moneta Health, Seattle, WA, USA; ^2^ Moneta Health, Jersey City, NJ, USA; ^3^ Moneta Health, Las Vegas, NV, USA; ^4^ Moneta Health, Dunedin, FL, USA; ^5^ Moneta Health, Cape Coral, FL, USA

## Abstract

**Introduction:**

Cognitive impairment affects 32% of adults over 65, impacting approximately 250 million people globally [1,2]. Cognitive rehabilitation is an effective intervention for dementia to preserve independence, delay institutional care and reduce caregiver burden [3]. However, access to rehabilitation is currently limited by a shortage of skilled providers, particularly in low resource and remote settings.

**Program Overview:**

Moneta Health is addressing this unmet need by delivering cognitive rehabilitation using an automated therapy delivery system. Patients receive personalized and interactive cognitive activities through a voice agent over the telephone focused on stimulating areas of cognitive deficit and teaching compensatory strategies. Automated calls are processed using artificial intelligence and reviewed by a speech‐language pathologist (SLP) who assigns therapy content, performs skilled analysis, assesses progress and provides weekly feedback to the patient by telephone.

**Program Results:**

In a cohort of 75 patients who completed Moneta's program (average age 73 ± 10, average MoCA score 20 ± 5), 59% had MCI and 33% had dementia. Patients completed 2.6 digital therapy calls (58 minutes) per week, and 2.3x more sessions overall than traditional outpatient therapy [4]. Patients' cognitive function significantly improved on average by 18% (*p* <0.001), compared to 13% for traditional outpatient therapy [4, 5]. Self reported quality of life also significantly improved on average by 11% (*p* <0.001) [6].

**Conclusion:**

Access to practical and personalized support is a critical component to helping older adults with cognitive impairment to remain independent in daily life. Moneta's AI‐powered program by telephone offers a scalable model for improving access to cognitive rehabilitation, with demonstrated real world outcomes.

**References**

[1] Manley et al., *JAMA Neurology*, 2022;79(12):1242‐1249.

[2] *Leaving No One Behind In An Ageing World: World Social Report*, United Nations, 2023.

[3] Kudlicka et al., Cognitive rehabilitation for people with mild to moderate dementia, *Cochrane Database of Systematic Reviews*, 2023.

[4] Compared to therapy outcomes data in the National Outcomes Measurement System of the American Speech‐Language‐Hearing Association for ages 50‐89, cognitive communication disorder, outpatient setting, accessed January 17, 2025.

[5] Functional communication measure (FCM) for cognitive function assessed by a SLP.

[6] Quality of Life in Neurological Disorders.